# Expand your body when you look at yourself: The role of the posture in a mirror exposure task

**DOI:** 10.1371/journal.pone.0194686

**Published:** 2018-03-23

**Authors:** Marta Miragall, Ernestina Etchemendy, Ausiàs Cebolla, Víctor Rodríguez, Carlos Medrano, Rosa María Baños

**Affiliations:** 1 Department of Personality, Evaluation and Psychological Treatment, University of Valencia, Valencia, Spain; 2 Department of Personality and Sociology, University of Zaragoza, Teruel, Spain; 3 CIBER Fisiopatología Obesidad y Nutrición (CIBEROBN), Instituto Carlos III, Madrid, Spain; 4 EduQTech, EUPT, University of Zaragoza, Teruel, Spain; 5 Aragon Institute for Health Research (IIS Aragón), University of Zaragoza, Zaragoza, Spain; University G d'Annunzio, ITALY

## Abstract

Mirror exposure (ME) is one of the main components of the treatment of patients with eating disorders symptomatology and it has shown its effectiveness in improving several outcomes (e.g., body dissatisfaction). However, the study as to what body posture should be adopted to maximize its effectiveness has been neglected. From embodied cognition and emotion theories, the adoption of an expansive (vs. contractive) body posture has been associated with positive changes in cognitive and emotional responses. The objective of this study was to analyze the effect of adopting an expansive (vs. contractive) posture before an ME task on body-related emotions and cognitions, as well as to analyze the possible moderator and mediator variables of these relationships. The sample was composed of 68 women (age: *M* = 21.74, *SD* = 3.12) with high scores on body dissatisfaction. Participants were randomly assigned to the expansive or contractive condition, where the openness of the arms/legs and the back position were manipulated. Posture was monitored by an electronic device and participants filled out several self-reported measures. ANCOVAs, moderation, mediation, and moderated mediated analyses were performed. Results showed that women in the expansive condition showed higher positive emotions after the ME. Moreover, exploratory analyses showed that adopting an expansive posture improved positive emotions, leading to improvements in negative emotions, body image satisfaction, and appraisal of the person’s own body. Psychological interventions should explore the value of holding an expansive posture before the ME in women with body dissatisfaction.

## Introduction

Body dissatisfaction has been defined as the negative subjective evaluation of one’s physical body, and it has been identified as a risk and maintenance factor for eating disorders (ED), such as anorexia and bulimia nervosa [1]. In fact, evidence indicates that individuals with ED symptomatology show negative emotional and cognitive responses, as well as changes in physiological responses, when they are exposed to their own bodies [2–4].

Mirror exposure (ME), based on the behavioral principles of exposure therapy, is a relevant component in the treatment of patients with body dissatisfaction. It generally consists of observing and describing each part of the person’s own body in a full-length mirror for a long period of time while wearing tight fitting clothes [5]. ME has been shown to be effective in improving several important outcomes (e.g., dissatisfaction, negative body-related emotions and cognitions, or other aspects of ED psychopathology) in clinical samples with ED (e.g., [6–8]), as well as in non-clinical samples (e.g., [9, 10]).

Several changes in the ME procedure have been applied varying, for instance, the descriptions individuals should make or the body part where the attention should be focused. Regarding descriptions, some studies have demonstrated the effectiveness of the ME procedure even without making them (that is, only observing the body) [4, 11], making non-judgmental or neutral descriptions [6, 12], or making positive descriptions [10]. Regarding where the attention is focused, Jansen et al. [13] showed that ME, focused on self-defined attractive or self-defined unattractive body parts, was equally effective, leading to improvements in body satisfaction. However, the study about which body posture should be adopted in the ME in order to maximize its effectiveness has been neglected.

Some studies have evidenced the link between the body posture and the body image concerns. Galeazzi et al. [14] have showed that the postural control during an ME task was significantly inversely related to the body image concerns in healthy participants. In this line, Forghieri et al. [15] found that when ED patients (compared to healthy participants) were exposed to their own body or images of thin ideal models, they showed greater postural destabilization. Moreover, Kolnes [16] described a disturbance in the posture besides the postural control in patients with anorexia nervosa, concluding that, among other characteristics, their posture tends to be stooped (e.g., the head and the shoulders are pulled forward).

Embodied cognition and emotion theories have highlighted the role of bodily states (e.g., sensor or motor states) in shaping cognition and emotion [17–19]. Specifically, the adoption of an expansive (vs. contractive) or an upright (vs. stooped) posture have been related to positive changes in emotional, cognitive, behavioral, and physiological responses, such as: increased feelings of power and risk-taking behavior, and hormonal changes associated with more dominance and less stress [20]; better nonverbal behavior (e.g., more confident) during a stressful job interview that led to a better overall performance [21]; higher self-esteem, more arousal, more positive mood, and less fear during a stressful speech task [22]; or more confidence in the self-attributes that individuals generated about their potential professional success [23]. Specifically, in the field of body dissatisfaction, Allen et al. [24] showed that women with high body dissatisfaction who were seated in an expansive (vs. contractive) posture attenuated the robust link between body dissatisfaction and restrained eating. Moreover, a recent Bayesian meta-analysis of preregistered studies found evidence for the effect of expansive postures (or “power posing”) on increasing self-reported felt power [25].

Despite the positive effects of expansive or upright (vs. contractive or stooped) postures on emotional and cognitive processes, no studies have analyzed the effect of adopting a specific posture in ME. Hence, the main objective of this study is to analyze the effect of adopting an expansive and upright (vs. contractive and stooped) posture for a short period of time before the ME on body-related emotions and cognitions: body- and eating-related negative thoughts during ME, as well as emotions, body image satisfaction (that is, the evaluative/affective experience related to one’s physical appearance), and appraisal of the person’s own body description after ME. Additionally, we developed an electronic device to monitor the posture before the ME task in order to ensure that women in each condition hold the appropriate posture objectively. The second aim of this study is to explore the more salient emotions felt by the participants while they were holding each posture. Moreover, the third aim is to analyze the role of the Body Mass Index (BMI) as a moderator on the effect of posture in changing body-related emotions and cognitions (negative thoughts during ME, as well as emotions, body image satisfaction, and the appraisal of the person’s own body after ME). Finally, the fourth and last aim is to explore possible mediators of the effects of posture on body-related emotions and cognitions after ME.

It is expected that the manipulation of the posture before the exposure to the person’s own body would act as a “positive state inductor” that would help to enhance the body-related emotions and cognitions in an ME task. This assumption is based on one of the mechanism of embodied cognition effects proposed by Körner et al. [26], in which bodily states may directly alter the individual’s feelings or information processing. Hence, the first hypotheses is that adopting an expansive (vs. contractive) posture would have positive effects on body-related emotions and cognitions (e.g., lower negative thoughts during ME, higher body image satisfaction after ME). The second hypothesis states that a greater percentage of women holding an expansive (vs. contractive) posture would feel positive emotions (e.g., self-confidence), whereas a greater percentage of women holding a contractive (vs. expansive) posture would feel negative emotions (e.g., insecurity). Moreover, the third hypothesis is that the BMI would moderate the effect of posture on changing body-related emotions and cognitions (e.g., women with lower BMI would achieve higher body image satisfaction after ME when adopting an expansive posture). This hypothesis is based on the meta-analysis by Stice [1], in which elevated BMI was identified as a risk factor for perceived pressure to be thin or for increased body dissatisfaction. Finally, no specific hypotheses are generated for the mechanisms of change that mediate the effect of posture on body-related emotions and cognitions due to the exploratory nature of the analyses.

## Methods

### Participants

The Ethics Committee at the University of Valencia (Spain) approved the study (approval number: H1477391858939). The sample was composed of 68 female students from the University of Valencia. The mean age was 21.74 (*SD* = 3.12), ranging from 18 to 35, and the average BMI was 22.60 (*SD* = 2.66), ranging from 18.20 to 29.61. The sample size was determined using G*Power [27], and a total of 34 participants per group were estimated to be included in the sample to detect a moderate effect size of *Cohen’s d* = 0.60 on the primary outcomes, taking into account the effect size found in other studies regarding the effect of posture manipulation on feelings of power, positive emotions, or self-esteem (e.g., [20, 22]), an alpha error of .05, and a statistical power of .80. The eligibility criteria for the present study included an age between 18 and 35 years, to reach a cut-off of ≥ 81 in the Body Shape Questionnaire (BSQ [28]) (a mild or more body dissatisfaction according to Cooper et al. [29]), and to have a BMI between 18.0 and 29.9 (to ensure that the body dissatisfaction is not due to excessive weight). The exclusion criteria were: (a) being currently under psychological treatment; (b) having a clinical history of ED; (c) being pregnant; and (d) reaching a cut-off ≥ 20 in the Eating Attitudes Test-26 (EAT-26 [30]) (to include participants without high ED symptomatology).

The screening questionnaires were completed by 491 women, but only 135 women met the participation criteria. They were invited to participate by phone, 23 participants did not respond to the call, 33 declined to participate in the experiment, and 11 did not came to the laboratory appointment. Finally, 68 women participated in the study and they were randomized. All participants signed informed consent documents before filling out the screening questionnaires and starting the experiment, in accordance with the Declaration of Helsinki.

## Measures and materials

### Demographic, medical, and psychological variables

An *ad-hoc* questionnaire was made to collect information regarding: age, weight, height, pregnancy, or psychological treatments.

### Trait body dissatisfaction: Body Shape Questionnaire (BSQ) [28]

This is a 34-item self-report questionnaire that assesses the dissatisfaction produced by one’s own body, the fear of gaining weight, self-devaluation due to physical appearance, desire to lose weight, and avoidance of situations in which physical appearance could attract the attention of others. Items are rated on a 6-point Likert scale, ranging from 1 (never) to 6 (always). The total score is the composite addition of the items (ranging from 34 to 204), and higher scores indicate higher body dissatisfaction in the past four weeks. According to Cooper et al. [29], the scores can be grouped in four categories or levels of concern: “no concern” (< 81), “mild concern” (81–110), “moderate concern” (111–140), and “extreme concern” (>140). The Spanish validation of the questionnaire was used [31]. This validation showed an adequate internal consistency in different non-clinical samples (α ranging from .95 to .97). In the present study, the internal consistency was also adequate (α = .84).

### Symptoms of ED: The Eating Attitudes Test-26 (EAT-26) [30]

This is a 26-item self-report questionnaire to be rated on a 6-point Likert scale ranging from 1 (never) to 6 (always). The total score is the composite addition of the items ranging from 0 to 78, that reflect the symptoms and concerns characteristic of ED. The scores for three subscales can also be calculated: (1) Dieting (items including avoidance of fattening foods and shape concerns); (2) Bulimia and Food Preoccupation (items including bulimic behaviors, and thoughts about food); and (3) Oral Control (items including self-control about intake and social pressure by the others to gain weight). The original authors of the questionnaire recommended the score ≥ 20 as a cut-off that indicates a high level of concern about dieting, body weight or problematic eating behaviors. The Spanish validation of the questionnaire was used [32]. This validation showed an adequate internal consistency in a non-clinical population (α = .86). In the present study, the internal consistency was adequate for the total score (α = .78).

### Body- and eating-related negative thoughts during the ME task: Thoughts Checklist (TCL) [33]

This checklist consists of 17 statements representing typical body-related negative thoughts (e.g., “I can’t look at myself in this mirror”) and eating-related negative thoughts (e.g., “I’ll go away and eat”) that people with ED have during the ME task. Items should be rated by the frequency of their occurrence (1 = thought did not occur; 6 = thought was there all the time), and a sum of negative cognitions frequency score is computed, ranging from 17 to 102. The Spanish version, translated by the authors, was used and it showed an adequate internal consistency (α = .82).

### Negative and positive emotions: Emotions’ Scale (ES)

A ES was used based on previous studies (e.g., [3, 4, 34]). Six negative emotions (shame, sadness, anger, insecurity, disgust, and anxiety) (negative ES) and two positive emotions (happiness and self-confidence) (positive ES) that may occur during the ME task were chosen by the authors (S1 Appendix). Participants had to rate to what extent they felt each emotion on a 7-point Likert scale (1 = not at all; 7 = completely) before and after the ME. Since the inter-item correlations for positive ES and negative ES were adequate (*r* > .20) according to Piedmont [35], two separate scores were calculated for positive and negative ES scores in order to reduce the probability of type I error. The internal consistency was adequate for negative ES across the administrations (α ranging from .78 to .88) and for positive ES at post-ME (α = .75), except for positive ES at pre-ME (α = .55).

### State body image: Body Image States Scale (BISS) [36]

This is a 6-item self-report questionnaire that measures the individuals’ evaluation and affect about their physical appearance (e.g., body image appraisal and satisfaction, as well as emotional experiences) at a particular moment in time: (1) satisfaction with one’s overall physical appearance, (2) satisfaction with one’s body size and shape, (3) satisfaction with one’s weight; (4) feelings of physical attractiveness; (5) current feelings about one’s looks relative to how one usually feels; and (6) evaluation of one’s appearance relative to how the average person looks. Items are rated on a 9-point bipolar Likert scale. The measure is the composite mean of the items, and higher scores reflect a more favorable body image satisfaction state. The BISS was acceptably internally consistent in the original validation. A Spanish version, translated by the authors, was used and it showed an adequate internal consistency across all administrations (α ranging from .81 to .87).

### Appraisal of the person’s own body descriptions

An *ad-hoc* questionnaire was developed (S1 Appendix). Participants had to appraise the adjectives that they used to describe their own body during the ME task as negative, neutral, or positive (e.g., “For me, that my hair is _____ is…”). They were asked to rate the two adjectives for 15 parts of their body (e.g., hair, buttocks) in an 11-point bipolar Likert scale (-5 = negative appraisal; 0 = neutral appraisal; +5 = positive appraisal). The total score was calculated by the mean from all the items. The internal consistency for this questionnaire was adequate (α = .79).

### Electronic device to monitor posture

**Fig 1:**
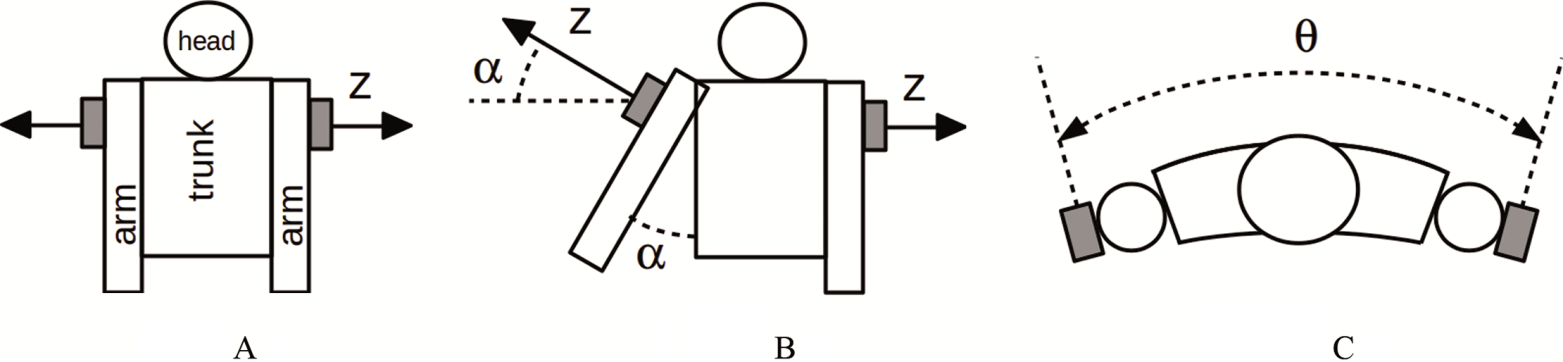
Schematic representation of the angles measured with the device. *Note*. Fig 1A = rest position (front view); Fig 1B = arm separated from trunk (front view); Fig 1C = chest expansion (top view).

An electronic measurement device was built around an inertial measurement unit (IMU MPU9255) connected to a microcontroller with a Bluetooth module. The set was mounted inside a small plastic box. An IMU includes an accelerometer, a magnetometer, and a gyroscope. It allows for the obtaining of the relative orientation of the IMU axes (x-y parallel to the IMU plane and z perpendicular to it) with respect to the earth’s axes (north, west, and vertical). In the experiment, the box was attached to the upper arm with an elastic strip. In the rest position, the IMU z-axis was approximately in the frontal human body plane (Fig 1A). From the rest position, the angle of the box with the vertical was measured (Fig 1B), as an estimation of the angle between the upper arm and the trunk (α). To reduce the number of parameters, and to facilitate posterior visualization, the mean angle of the left and right arms was obtained. In addition, if the chest was expanded by a movement of the shoulder blades, the devices were rotated and the angle between them on the horizontal plane was also measured (θ) (Fig 1C). Both, α and θ were calibrated at the rest position, so that those initial angles were subtracted from the posterior measurements. During the experiments, the sensor sent values at 1 Hz to a PC with a Bluetooth port.

### The ME task

The ME task carried out in this study was based on the procedure followed by Luethcke et al. [10] and Delinsky et al. [12]. Participants had to observe their own bodies in a three-way mirror and describe different parts of their body. To do so, participants had to make two comments on 15 parts of their body in the following order: hair, skin, eyes, nose, mouth, neck, arms, chest, waist, hips, buttocks, thighs, knees, calves, and feet. An audio file was recorded, including the 15 parts of the body, with the following standardized instructions: (1) Focus on your ______ (e.g., hair); (2) Make two comments about your ______ (e.g., hair). The participants must focus their attention for 10 seconds on the specific part of the body, and then, they had up to 25 seconds to make the two descriptions of the specific part of the body. They were asked not to focus on other parts of the body until the next instruction was given. The task lasted 10 minutes approximately.

Regarding the descriptions, the only instruction that was given to the participants is that the description of their body should be related to the physical appearance (e.g., shape, length, width, symmetry, coloring). Moreover, the researcher recommended to the participants that they should make the comments while keeping in mind that they could not be seen and that their image would be drawn according to their descriptions. Because of that, they should avoid evaluative comments or subjective terms, such as “beautiful” or “ugly”, or “I like it” or “I don’t like it”. The researcher checked the complete understanding of the task before starting, requesting an example of the description of their hand (a part not included in the task).

Participants should have a complete view of their own bodies in the three-way mirror. Thus, they had to remain on a mark placed on the floor in front of the mirror. However, they could move to get a better view of some parts of their own body (e.g., to look at their buttocks). Before beginning the task, to make the own view of the body similar for all the participants, they were requested to put on grey cotton leggings and a grey cotton short-sleeve t-shirt in a size that best fitted the participant.

A researcher remained in the room while the participant did the ME task, but the researcher was out of the participant’s view, not looking at her. The researcher wrote down all the descriptions that the participants made about their body parts. The researcher only spoke to the participants if they skipped the comments they should make. Moreover, the researcher reminded them the kind of description that they could make if they got stuck in a body part or if they used evaluative comments.

### Procedure

The sample was recruited from both the Psychology and the Speech Therapy degree programs at the University of Valencia, where researchers invited the students to participate in a study related to “body image and the validation of body motion sensors”. Notices were also placed on several bulletin boards in the Faculty of Psychology. Once the participants expressed a wish to participate, they filled out the informed consent and the screening questionnaires (*ad-hoc* questionnaire, BSQ and EAT-26). Only participants who met the inclusion and exclusion criteria were invited to participate by a phone call or e-mail. They were informed that it was mandatory not to eat or perform intense physical activity in the 2 hours before coming to the laboratory in order to standardize feelings of fatness, and they were informed that the researcher was going to ask them to change their clothes during the experiment.

**Fig 2:**
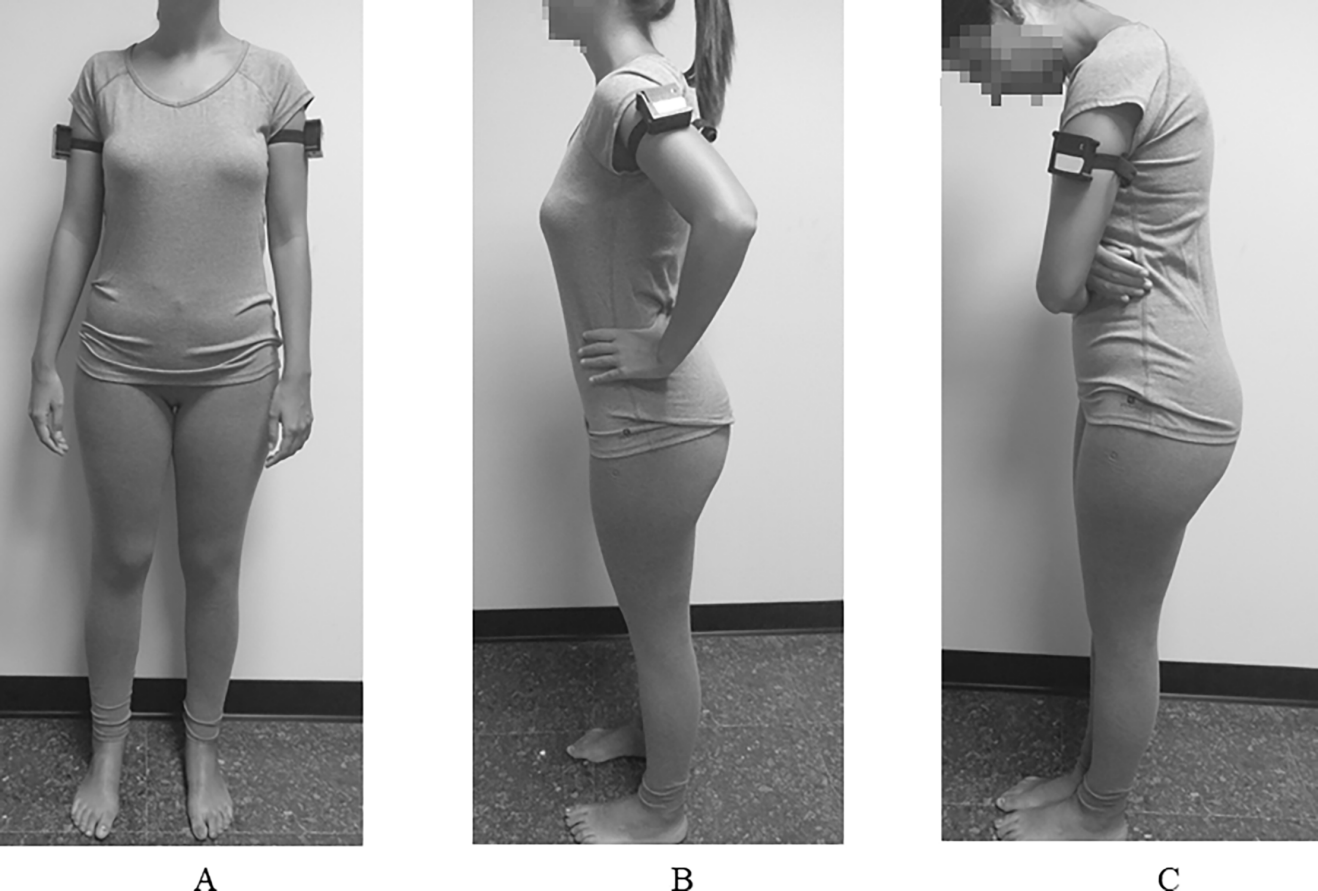
Postures adopted during the experiment. *Note*. Fig 2A = Neutral posture (for calibrating); Fig 2B = Expansive posture; Fig 2C = Contractive posture.

Participants were randomly assigned to the expansive (*n* = 34) or contractive (*n* = 34) condition using the Random Allocation Software 2.0 [37]. No participants were excluded. When participants came to the laboratory, they filled out the ES and the BISS. Then, the researcher left the room to allow the participants to change their clothes. Next, the electronic measurement devices were placed on both upper arms, and the researcher asked the participants to stay in a neutral posture (upright, with arms extended and close to the trunk) for one minute to calibrate the body (Fig 2A). Subsequently, the ME task was explained to the participants, but the researcher told the participants that it was necessary to do a new calibration of the body sensor before starting the task (to cover the main objective of the study).

The posture was manipulated following the previous studies [20, 21, 38], where the expansiveness or openness (keeping arms and legs open or closed), as well as the position of the back (upright or slumped) were manipulated. The decision to make the participants hold the posture only before the ME (not during) and for a short period of time (2 minutes) is based on the study by Carney et al. [20] and the conclusions of the review by Carney et al. [39], that suggested that the length of time that the posture is adopted should be carefully considered because it seems to be an important moderator variable responsible for the contradictory results found in the effects of posture (e.g., holding some postures for a long time might cause discomfort or habituation). In addition, Ortega-Roldán et al. [40] found that women with high body dissatisfaction (compared to women with low body dissatisfaction) that were exposed to photographs of their own body in model postures (upright position with one hand on the waist and the other extended, and one knee slightly flexed) compared to neutral postures (upright position with arms extended and close to the trunk) showed less pleasure, more negative and ugliness feelings, and an increased startle response (a reflex that is potentiated when processing stimuli associated with negative affect). Moreover, the decision to manipulate the women’s backs as well as to expand the body, is based on the positive results that the upright postures have shown (e.g., [22, 23]).

The experimenter gave the instructions verbally, modelled the posture, and showed a photo of the posture to the participants. In the expansive condition, participants had to adopt an expansive, upright, and open posture (standing up, with the chest out, shoulders back and the back straight, chin parallel to floor, arms at hips, and legs slightly open) (Fig 2B). In contrast, in the contractive condition, participants had to adopt a contractive, stooped, and closed posture, taking up less space (standing up, with stooped back, dropping the rib cage with shoulders forward, neck slightly down, and hands and legs together and intertwined) (Fig 2C).

After adopting the posture for 2 minutes, participants were placed in front of the mirror adopting a free posture. Once the task was finished, they completed the TCL, the ES, the BISS, and the questionnaire about the appraisal of the person’s own body descriptions. We also requested the participants to choose one emotion (self-confidence, aplomb, dignity, arrogance, pride, submissiveness, sadness, insecurity, and dominance) that best reflected their experience in the adopted posture. Moreover, they were weighed on a weighing scale. At the end of the experiment, we interrogated each participant to make sure that they had not discovered the hypothesis of the study. No participants guessed the deception involved in the posture manipulation or the connection between the effects of posture on the body-related emotions and cognitions. The experiment lasted 45 minutes approximately and participants were given €5 for their participation.

### Data analyses

**Fig 3:**
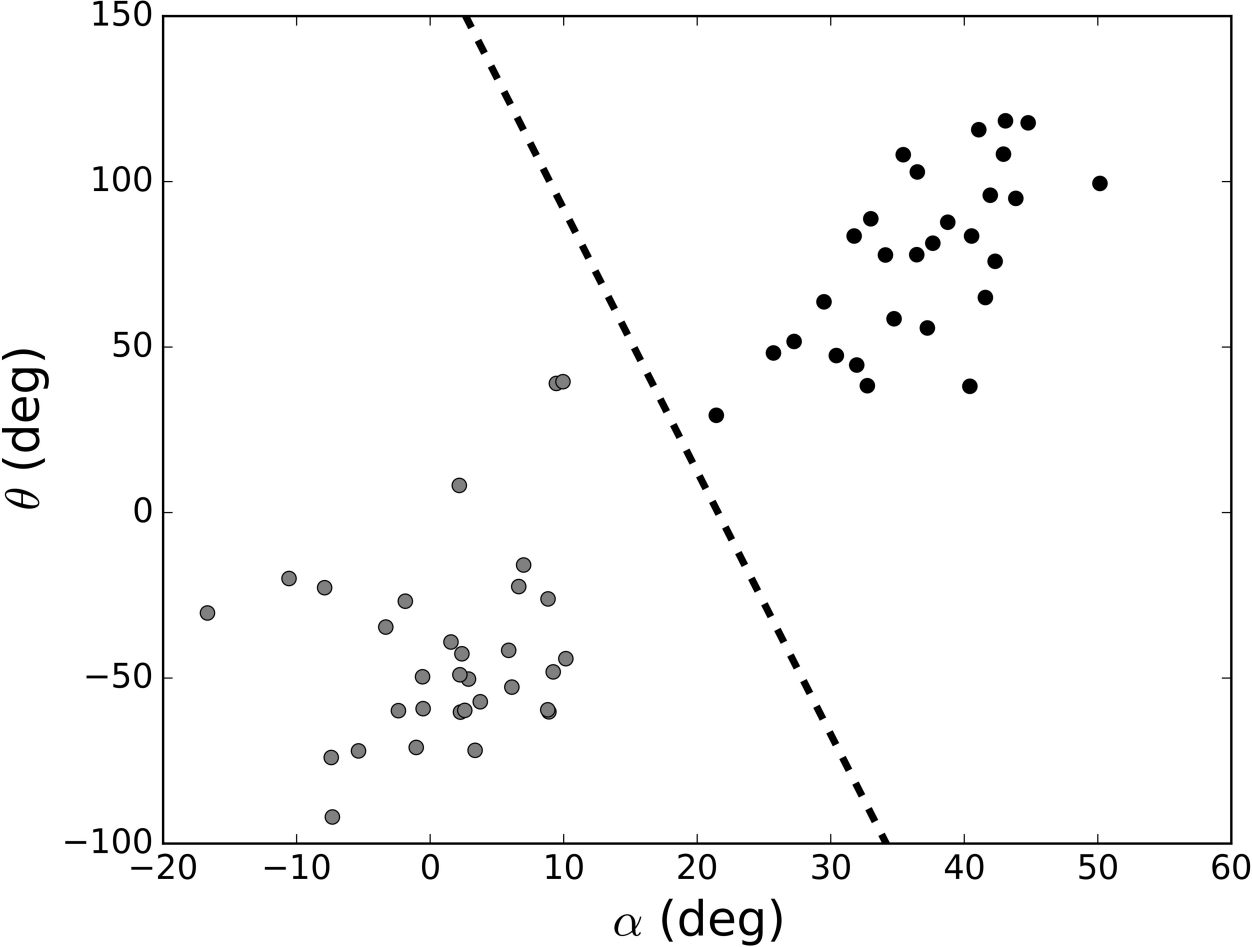
Mean value of θ (the angle between shoulder blades) and α (angle between the upper arm and the trunk) during body posture manipulation. *Note*. Grey circles: contractive condition; dark circles: expansive condition. The dashed line separates the two classes. deg = degrees.

Statistical analyses were performed using the SPSS v.24 software. Regarding the posture manipulation (measured by an electronic device), after the manipulation of the posture for 2 minutes, the mean values of α and θ were represented (Fig 1B and 1C). One of the points was removed as an outlier. It was visually apparent that these angles were different depending on the kind of posture adopted. To characterize it with a single value, a linear discriminant analysis was performed. The line that separated the two classes was obtained and the signed distance to it was used as a feature related to expansive posture (Fig 3). Moreover, an independent-sample t-test was performed to test whether there were significant differences in this value. Due to problems with the electronic device connection, calculations were made with 29 and 31 participants in the expansive and contractive condition, respectively.

Regarding the analyses in self-measures, firstly, descriptive statistics and four independent-samples t-test were performed to verify that there were no significant differences in age, BMI, BSQ, EAT-26 at baseline across conditions. Secondly, eleven ANCOVAs (with the condition as between-factor and baseline scores as covariates) and two independent-samples t-test were performed to check the effects of the posture on BISS, negative and positive ES, TCL, and appraisal of the person’s own body description.

Thirdly, to analyze the differences in the percentage of emotions felt in each condition, a chi-square test was performed, using Monte Carlo with 10,000 samples and a 99% of level of confidence. When the absolute value of the Adjusted Standarized Residual was greater than 1.96, the relationship between the different categories was considered significant, and the sign indicated the type of relationship between the categories.

Finally, five moderation analyses and three mediation analysis were performed using the procedure described by Hayes [41] from the PROCESS macro (version 2.16), choosing model 1 and 4, respectively. Pre-ME scores were entered as covariates of post-ME scores for negative and positive ES, and BISS models. The expansive condition was coded as “1” and the contractive condition was coded as “2”. Thus, a negative relationship between condition and positive ES, BISS, and appraisal of the person’s own body description, and a positive relationship between condition and negative ES, and TCL meant that participants that adopted an expansive posture showed greater improvements in these measures. All regression coefficients were reported in unstandardized form as *b*-values.

Firstly, moderation analyses were carried out to examine whether the relationships between the condition and the post-ME scores of TCL, negative and positive ES, BISS and, the appraisal of the person’s own body description, were moderated by BMI. Tests of significance (*p* < .05) or a confidence interval (not including zero) in the interaction “condition x BMI” answered whether the BMI moderated the effect of condition on post-ME scores. We examined the conditional effect of condition on the post-ME scores at medium (the mean), low (-1 *SD*), and high (+1 *SD*) levels of BMI with the pick-a-point approach (or analysis of the simple slopes). Secondly, as significant differences were only found in positive ES between conditions after the ME, simple mediation analyses were performed to test whether the effect of condition on post-ME scores of negative ES, BISS and appraisal of the person’s own body description were mediated by the change in positive ES. Change in positive ES was calculated using pre-ME scores and post-ME scores (e.g., Change = post-score–pre-score), where positive values for change in positive ES reflected an improvement. Bias-corrected bootstrap 95% confidence intervals (CIs) based on 5,000 samples were used to assess the indirect effects. CI that did not include the zero-value indicated a significant indirect effect, meaning that the effect of the condition on the post-ME scores was mediated by the change in positive ES. Additionally, to analyze the combined influence of the BMI and change in positive ES in the relationship between posture and post-ME scores of body image satisfaction, an exploratory moderated mediation model (“model 5”) was performed. This combined the effect of condition on BISS post-ME scores indirectly through the change in positive ES, but also the direct the effect of posture on BISS post-ME scores depending on BMI.

## Results

### Checking differences in age, BMI, BSQ and EAT-26

Descriptive statistics are shown in Table 1. There were no significant differences between conditions on age, *t*(51.94) = 1.09, *p* = .280, *Cohen’s d* = 0.27; BMI, *t*(66) = 1.48, *p* = .144, *Cohen’s d* = 0.36; BSQ, *t*(66) = 1.40, *p* = .165, *Cohen’s d* = 0.34; and EAT-26, *t*(66) = 0.26, *p* = .796, *Cohen’s d* = 0.06.

**Table 1:** Descriptive statistics for age, BMI, BSQ, and EAT-26.

	Expansive condition (*n* = 34) *M (SD)*	Contractive condition (*n* = 34) *M (SD)*
**Age (years)**	22.15 (3.84)	21.32 (2.16)
**BMI**	23.07 (2.43)	22.13 (2.83)
**BSQ**	107.00 (16.95)	101.38 (16.06)
**EAT-26**	7.79 (5.34)	7.47 (4.95)

*Note*. BMI = Body Mass Index; BSQ = Body Shape Questionnaire; EAT-26 = The Eating Attitudes Test-26.

### Checking the posture manipulation before the ME

Significant differences were found between conditions on the adopted posture before the ME, *t*(58) = 8.60, *p* < .001, *Cohen’s d* = 2.22. Participants in the expansive condition achieved a higher expansive posture (*M* = 18.43, *SD* = 19.27) than participants in the contractive condition (*M* = -20.84, *SD* = 16.05) according to the α and θ angles.

### Effects of posture on TCL, negative and positive ES, BISS, and appraisal of the person’s own body descriptions

Descriptive statistics, ANCOVAs, and independent-samples t-test results for TCL, negative and positive ES, BISS, and appraisal of the person’s own body descriptions are shown in Table 2. A main effect of condition for the total score of positive ES was found. Pairwise comparisons showed that participants in the expansive condition showed more positive emotions than participants in the contractive condition (*p* = .006). Separately, ANOVAs for self-confidence and happiness also showed a main effect of condition. Pairwise comparisons showed that participants in the expansive condition showed more self-confidence and happiness than participants in the contractive condition (*p* = .015 and *p* = .029, respectively). However, there were no main effects of condition for BISS and negative ES (insecurity, anxiety, disgust, shame, sadness, anger, and total score). In the same line, significant differences were not found between the expansive and contractive conditions on TCL and the appraisal of the person’s own body comments.

**Table 2:** ANCOVAs and independent-samples t-test results and values for TCL, negative and positive ES, BISS, and appraisal of the person’s own body descriptions.

	Expansive condition (*n* = 34)		Contractive condition (*n* = 34)		ANCOVA ^a^ / t-Student		
	*Pre*	*Post*	*Pre*	*Post*	*F / t*	*p*	*η*_*p*_^*2*^ */ Cohen’s d*
	*M(SD)*	*M(SD)*	*M(SD)*	*M(SD)*			
**TCL**	-	31.50 (8.53)	-	33.41 (8.91)	*t*(66) = -0.90	.369	*d* = -0.22
**Negative ES (total)**	1.89 (0.69)	2.12 (1.08)	2.28 (0.87)	2.61 (1.24)	*F*(1,65) = 0.34	.551	*η*_*p*_^*2*^ = .01
Insecurity	2.79 (1.39)	2.88 (1.49)	3.56 (1.33)	3.68 (1.68)	*F*(1,65) = 0.66	.419	*η*_*p*_^*2*^ = .01
Anxiety	2.00 (1.13)	2.09 (1.31)	2.77 (1.35)	2.62 (1.78)	*F*(1,65) = 0.41	.524	*η*_*p*_^*2*^ = .01
Disgust	1.18 (0.63)	1.47 (1.21)	1.38 (0.74)	1.77 (1.02)	*F*(1,65) = 0.42	.518	*η*_*p*_^*2*^ = .01
Shame	2.27 (1.19)	2.47 (1.66)	2.32 (1.34)	2.88 (1.75)	*F*(1,65) = 1.20	.278	*η*_*p*_^*2*^ = .02
Sadness	1.79 (1.12)	2.15 (1.23)	1.94 (1.30)	2.47 (1.50)	*F*(1,65) = 0.68	.412	*η*_*p*_^*2*^ = .01
Anger	1.29 (0.52)	1.68 (1.36)	1.68 (1.27)	2.27 (1.50)	*F*(1,65) = 1.30	.258	*η*_*p*_^*2*^ = .02
**Positive ES (total)**	4.90 (0.89)	4.66 (1.13)	4.85 (0.92)	3.97 (1.24)	*F*(1,65) = 8.10	.006	*η*_*p*_^*2*^ ***=*** .11
Self-confidence	4.91 (1.08)	4.74 (1.36)	4.68 (1.04)	3.85 (1.52)	*F*(1,65) = 6.29	.015	*η*_*p*_^*2*^ ***=*** .09
Happiness	4.88 (1.07)	4.59 (1.16)	5.03 (1.14)	4.09 (1.29)	*F*(1,65) = 4.98	.029	*η*_*p*_^*2*^ ***=*** .07
**BISS**	5.02 (1.06)	4.43 (1.38)	4.97 (1.22)	4.20 (1.38)	*F*(1,65) = 0.74	.392	*η*_*p*_^*2*^ ***=*** .01
**Appraisal of the person’s own body descriptions**	-	0.33 (1.27)	-	0.09 (0.92)	*t*(65) = 0.86	.393	*d* = 0.21

*Note*.

^a^ All ANCOVAs were adjusted for baseline scores.

TCL = Thoughts Checklist; ES = Emotions’ Scale; BISS = Body Image States Scale.

### Differences in the percentage of emotions felt in each condition

The crosstabs of the percentage of feelings in each condition are shown in Table 3. Results showed that the feelings differed significantly depending on the adopted posture, χ^2^(7, N = 68) = 29.76, *p* < .001, Cramer’s V = 0.66. Participants who adopted an expansive posture experienced more “self-confidence” than participants who adopted a contractive posture (Adjusted Standarized Residuals = 3.8). Moreover, participants who adopted a contractive posture experienced more “submissiveness” (Adjusted Standarized Residuals = 3.4) and “insecurity” (Adjusted Standarized Residuals = 2.4) than participants who adopted an expansive posture.

**Table 3:** Crosstab of the percentage of emotions in each condition.

		Expansive condition	Contractive condition	Total
**Self-confidence**	Count	20	5	25
	Expected count	12.5	12.5	25.0
	%	80%	20%	100%
	ASR	3.8	-3.8	
**Aplomb**	Count	5	3	8
	Expected count	4.0	4.0	8.0
	%	62.5%	37.5%	100%
	ASR	0.8	-0.8	
**Dignity**	Count	1	0	1
	Expected count	0.5	0.5	1.0
	%	100%	0%	100%
	ASR	1.0	-1.0	
**Arrogance**	Count	1	0	1
	Expected count	0.5	0.5	1.0
	%	100%	0	100%
	ASR	1.0	-1.0	
**Pride**	Count	2	0	2
	Expected count	1.0	1.0	2.0
	%	100%	0%	100%
	ASR	1.4	-1.4	
**Submissiveness**	Count	0	10	10
	Expected count	5.0	5.0	10.0
	%	0%	100%	100%
	ASR	-3.4	3.4	
**Sadness**	Count	0	2	2
	Expected count	1.0	1.0	2.0
	%	0%	100%	100%
	ASR	-1.4	1.4	
**Insecurity**	Count	5	14	19
	Expected count	9.5	9.5	19.0
	%	26.3%	73.7%	100%
	ASR	-2.4	2.4	
**Dominance**	Count	0	0	0
	Expected count	-	-	-
	%	0%	0%	0%
	ASR	-	-	-
**Total**	Count	34	34	68

*Note*. ASR = Adjusted standardized residuals.

### BMI as moderator: Does the BMI moderate the effect of posture on the TCL, negative and positive ES, BISS, and appraisal of the person’s own body description?

Moderation analyses showed that the BMI moderated the effect of condition on the BISS post-ME scores, but not on the TCL, *F*(1,64) = 0.11, *p* = .745; the negative ES post-ME scores, *F*(1,63) = 0.31, *p* = .583; the positive ES post-ME scores, *F*(1,63) = 0.78, *p* = .381; or on the appraisal of the person’s own body description, *F*(1,63) = 0.33, *p* = .566. In the case of the BISS, the overall model explained 63.34% of the variance in the BISS post-ME scores, and it was significant, *F*(4,63) = 46.99, *p* < .001. The interaction between condition and BMI was significant, *F*(1,63) = 5.86, *p* = .018, meaning that BMI was a moderator of the effect of the condition on the BISS post-ME scores, accounting for 2.04% of the variance. Analysis of simple slopes showed that there was a significant negative relationship between condition and the BISS post-ME scores when the BMI was “low”, *b* = -0.66, 95% CI [-1.22, -0.11], *t* = -2.39, *p* = .020. Participants in the expansive condition (compared to the contractive condition) with lower BMI achieved higher scores in body image satisfaction after the ME task.

### Positive emotions as mediators: Does the posture influence negative ES, BISS and appraisal of the person’s own body description through the change in positive ES?

**Fig 4:**
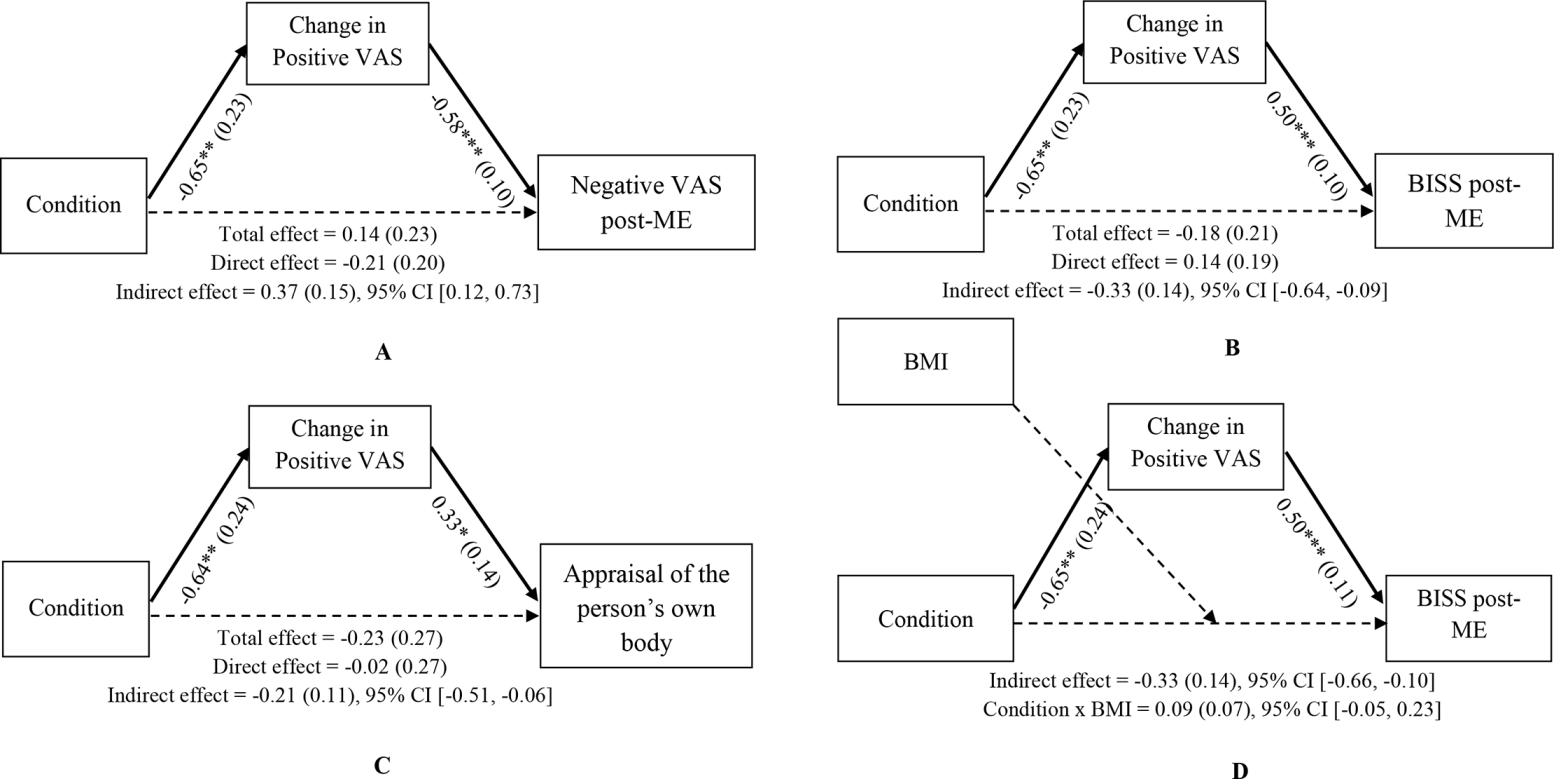
**Simple mediation analyses (Fig 4A, 4B and 4C) and moderated mediation analysis (Fig 4D).** *Note*. All coefficients represent unstandardized regression coefficients (standard errors in parenthesis). Pre-ME scores of negative ES and BISS are entered as covariates in the negative ES and BISS models. * *p* < .05; ** *p* < .01; *** *p* < .001. ME = Mirror Exposure; BISS = Body Image States Scale; ES = Emotions’ Scale. BMI = Body Mass Index.

Unstandardized regression coefficients, standard errors in parenthesis, and confidence intervals of the direct, total, and indirect effects are shown in Fig 4A, 4B and 4C. The indirect effects for all the models were significant, implying that the change in positive ES mediated the relationship between condition and the post-ME scores of negative ES, BISS, and appraisal of the person’s own body. These results meant that participants who adopted an expansive posture (compared to a contractive posture) had more positive changes in positive emotions after the ME task, leading to less negative emotions, and more positive scores in body image satisfaction and the appraisal of their own body descriptions.

### Moderated mediation: How does the change in positive ES and BMI interact with the effect of posture on the BISS?

Unstandardized regression coefficients, standard errors in parentheses, and confidence interval of the indirect effects are shown in Fig 4D. In this model, the BMI did not moderate the direct effect of posture on BISS post-ME (independent of the influence of posture on BISS post-ME scores through change in positive ES), as the interaction between condition and BMI was not significant, *b* = 0.09, *SE* = 0.07, *t* = 1.33, *p* = .190, 95% CI [-0.05, 0.23], and any conditional direct effect was significant at any value of the moderator: “low BMI”, *b* = -0.20, *SE* = 0.25, *t* = -0.82, *p* = .417, 95% CI [-0.69, 0.29]; “medium BMI”, *b* = 0.05, *SE* = 0.19, *t* = 0.25, *p* = .807, 95% CI [-0.33, 0.42 ], “high BMI”, *b* = 0.29, *SE* = 0.28, *t* = 1.04, *p* = .301, 95% CI [-0.27, 0.85]. However, the indirect effect remained significant, *b* = -0.33, *SE* = 0.14, 95% CI [-0.66, -0.10]. Hence, the posture exerted its effect on body image satisfaction after the ME indirectly through the change in positive emotions, but not directly when the BMI was introduced as moderator.

## Discussion

The main objective of this study was to analyze the effect of adopting an expansive (vs. a contractive) posture for 2 minutes before an ME task on body- and eating-related negative thoughts during the ME, as well as on positive and negative emotions, body image satisfaction, and appraisal of the person’s own body description after the ME. Moreover, the secondary objectives were to explore the differences in the percentage of emotions felt by the women while they were holding each posture, as well as to analyze the possible moderator and mediator variables of the effect of the posture on the body-related emotions and cognitions.

Firstly, the successful manipulation of the posture was checked. The electronic device showed that there were significant differences in the expansiveness of the body between both conditions. Regarding the effects of posture on the body-related emotions and cognitions after the ME, results showed that only the level of positive emotions after the ME changed, as women who adopted an expansive posture showed more positive emotions (a combination of happiness and self-confidence). This is in line with other studies that have shown that expansive or upright postures generate changes in positive emotions (e.g., [22, 42]). Moreover, it has corroborated the conclusion drawn by Jonas et al. [43] about the necessity of analyzing the effect of expansive posture on other variables beyond the “feelings of power”, which has been one of the most studied variables related to the effects of this posture up to now. Nevertheless, significant differences were not found between condition on body- and eating-related negative thoughts during the ME, nor negative emotions, body image satisfaction, and appraisal of the person’s own body description after the ME. Hence, the first hypothesis was partially supported. In the same line, it was found that a greater percentage of women experienced self-confidence in the expansive condition, while it was found that a greater percentage of women experienced submissiveness and insecurity in the contractive condition. Hence, the second hypothesis was supported. Regarding the third hypothesis, it was partially supported because the BMI only moderated the relationship between the effect of posture on body image satisfaction, but not the rest of body-related emotions and cognitions. Although the moderation did not emerge in the rest of the outcome variables, it is in line with the meta-analysis of Stice [1] who found an association between BMI and body dissatisfaction. Thus, women with lower BMI achieved a better body image satisfaction after the ME when they had adopted an expansive posture.

In addition, the exploratory mediation analyses showed that the change in positive emotions after the ME acted as a mechanism of change in the effect of adopting an expansive (vs. contractive) posture on negative emotions, body image satisfaction, and appraisal of the person’s own body. It suggests that a preparatory expansive posture before the ME acts as an inducer of positive emotions, which helps to re-evaluate the body in a more positive way. This result contradicts the inconsistent effects of the “power poses” (or expansive postures) found in recent replications of the study by Carney et al. [20] (e.g., [44, 45]) or the results of an analysis of different studies made by Simmons et al. [46] that concluded that there is a lack of empirical support for the effects of “power poses”. As De Zabala et al. [47] hypothesized, the inconsistent effects of “power poses” may be related to the lack of manipulation of the lift of the spine and the expansion of the chest. In fact, they compared open and closed front body yoga postures (e.g., emphasizing the lift of the spine and the expansion of the chest) and the expansive and contractive postures used in other studies such as Carney et al. [20], showing that yoga postures (as compared to “power poses”) improved self-esteem through increasing the subjective sense of energy and empowerment.

Although yoga postures were not adopted in this study, some manipulations that were not considered in other expansive-contractive postures studies (e.g., [20, 21, 44, 45]) were emphasized, such as the position of the back and the chest. Thus, in this case, the position of the back and the chest as well as the expansiveness of the body seem to improve the body-related emotions and cognitions after the ME driven by the increase in happiness and self-confidence. Because of that, it is important to determine what adopted posture is effective to change a specific psychological variable, as other postures may have no effects or contrary effects in the cognitive and emotional response in the ME because they are driven by other mechanisms (e.g., dominance in the case of expansive postures without manipulating the back or chest).

Thus, the role of positive emotions as a mechanism of change in the effect of posture on body-related emotions and cognitions seems highly relevant. The exploratory moderated mediation analysis showed that when the effect of the BMI and change in positive emotions were introduced in the same model, the BMI was no longer relevant in explaining the changes in body image satisfaction, as the conditional direct effect of the posture on body image satisfaction depending on BMI disappeared. Results suggest that when an expansive posture is adopted, it generates such a strong change in positive emotions that the BMI of the women does not have any influence on the increase of the body image satisfaction after the ME.

The limitations of the current study should be noted. First, the study has been done with a non-clinical sample, so the results are not completely generalizable to other populations, and it is especially relevant whether this response is replicable in ED samples. Although several ME-related studies have recruited non-clinical samples to analyze its effects (e.g., [10]), the study by Trentowska et al. [48] supports the view that non-clinical samples react to ME at a much lower level and with different emotional changes than patients with ED (e.g., bulimia nervosa). Second, a control condition was not used because it was intended to analyze the differences between an expansive and a contractive posture. However, a third condition, with a natural posture without manipulating the body could help to resolve whether a preparatory expansive posture has superior positive effects in the cognitive and emotional response in the ME over a preparatory natural posture. Third, although the participants should look at different parts of their body, it was not possible to verify whether they were looking at the specific part or if they avoided some parts. Forth, physiological measures (e.g., skin conductance, heart rate) were not used. It would have been interesting to have these measures in order to analyze the role of posture in these responses. Servián-Franco et al. [49] found that women with high body dissatisfaction showed a reduced physiological response compared to women with low body dissatisfaction. The authors suggested that this low response could be related to a passive (vs. an active) coping style that could interfere in the efficacy of the ME. Because of this, future studies should analyze the physiological effects of the posture in the ME, and explore the possible relation with an active or passive style of coping during the exposure. In addition, it would have been interesting to assess the posturographic destabilization of the women’s body to analyze whether a preparatory posture generates differences between conditions on postural destabilization.

Moreover, more research is needed in order to analyze whether the posture improves the outcomes in the ME treatment of patients with ED. According to the approaches that try to explain the mechanisms underlying the exposure therapy, such as the habituation-based models (e.g., “emotional processing model”; [50]), there is a negative response that makes the exposure work. Trentowska et al. [34] concluded that the process of change in the ME related to the improvement in body image in patients with ED is comparable to fear habituation processes. In the same line, Trentowska et al. [48] found a cognitive and emotional (although not physiological) habituation within and between ME sessions in patients with ED. Nevertheless, Díaz-Ferrer et al. [9] found that the subjective discomfort was maintained in the first session without evidence of within-session habituation process, making it comparable to the inhibitory learning model of extinction [51, 52]. Hence, it is important to test whether the adoption of an expansive posture has negative consequences for the therapy, impeding the necessary negative response claimed by some models (e.g., [50]), or, in contrast, it has positive consequences for the therapy through the increase in positive emotions, making within-session habituation easier (e.g., increasing the view of one’s ability to actively cope with the exposure), making between-session habituation easier (e.g., increasing the adherence to several sessions of exposure therapy), or making easier the development of new non-threating associations within sessions, competing with the original negative response.

Hence, psychological interventions should explore the convenience of holding an expansive posture before the ME in women with body dissatisfaction. In fact, some authors have highlighted the necessity to determine which body manipulations could be beneficial to modify the cognitions and emotions in therapy [53]. These authors proposed the embodied cognition area as a field that has clear clinical applications as a “wise intervention”, a kind of intervention outlined by Walton [54].

In conclusion, this study shows the role of preparatory postures on changing the body-related emotions and cognitions after being exposed to the person’s own body in an ME task in women with high body dissatisfaction. The main finding of this study is that more positive emotions are achieved when an expansive (vs. contractive) posture is adopted for 2 minutes before an ME task. Moreover, the increase in positive emotions constitutes the mechanism of change of the effect of adopting an expansive posture on reducing negative emotions, and increasing body image satisfaction and the appraisal of the person’s own body after the ME. In line with embodied cognition and emotion theories, the results of this study suggest that the adoption of an expansive posture seems to be a promising therapeutic strategy and that it should continue to be explored due to its positives effects on the cognitive and emotional response in the ME. However, more research is needed in order to establish how, when, or for whom the adoption of an expansive posture is effective in the exposure to the person’s own body.

## Supporting information

S1 Appendix Questionnaires used in the present study (Spanish and English versions). (DOCX)
