# Supplementary material for: Expand your body when you look at yourself: The role of the posture in a mirror exposure task
Source: PLoS One. 2018 Mar 23;13(3):e0194686. doi: 10.1371/journal.pone.0194686 (PMC5865731; doi:10.1371/journal.pone.0194686)
Supplement: S1 Appendix — (DOCX) [file pone.0194686.s001.docx]

**S1 Appendix. Questionnaires used in the present study (Spanish and English versions).**

The questionnaires used in this study that have been published previously are cited in this document:

1. **Body Shape Questionnaire (BSQ)**

- **English version:** Cooper PJ, Taylor MJ, Cooper Z, Fairburn CG. The development and validation of the Body Shape Questionnaire. Int J Eat Disord. 1987; 6: 485–94. doi: 10.1002/1098-108X(198707)6:4<485::AID-EAT2260060405>3.0.CO;2-O
- **Spanish version:** Raich RM, Mora M, Soler A, Ávila C, Clos I, Zapater I. Adaptación de un instrumento de evaluación de la insatisfacción corporal [Adaptation of an instrument for assessing body dissatisfaction]. Clínica y Salud. 1996; 7(1), 51–66. Available from: http://www.copmadrid.org/webcopm/publicaciones/clinica/1996/vol1/arti4.htm

1. **The Eating Attitudes Test-26 (EAT-26)**

- **English version:** Garner DM, Olmsted MP, Bohr Y, Garfinkel PE. The eating attitudes test: Psychometric features and clinical correlates. Psychol Med. 1982; 12(4): 871–8. doi: 10.1017/S0033291700049163
- **Spanish version:** Gandarillas A, Zorrilla B, Sepúlveda AR, Muñoz R. Trastornos del comportamiento alimentarios: Prevalencia de casos clínicos en mujeres adolescentes de la Comunidad de Madrid [Eating behavior disorders: Prevalence of clinical cases in adolescent women in the Community of Madrid]. Documentos Técnicos de Salud Pública, Instituto de Salud Pública. 2003; 85. Available from: http://www.madrid.org/cs/Satellite?blobcol=urldata&blobheader=application%2Fpdf&blobheadername1=Content-disposition&blobheadername2=cadena&blobheadervalue1=filename%3DDT85_prevalencia+de+casos.pdf&blobheadervalue2=language%3Des%26site%3DPortalSalud&blobkey=id&blobtable=MungoBlobs&blobwhere=1352853150802&ssbinary=true

1. **Thoughts Checklist (TCL)**

- **English version:** Cooper MJ, Fairburn CG. Thoughts about eating, weight and shape in anorexia nervosa and bulimia nervosa. Behaviour Research and Therapy. 1992; 30(5): 501–511. doi: [10.1016/0005-7967(92)90034-E](https://doi.org/10.1016/0005-7967(92)90034-E)
- **Spanish version:** Translated by the authors.

1. **Body Image States Scale (BISS)**

- **English version:** Cash TF, Fleming EC, Alindogan J, Steadman L, Whitehead A.  Beyond body image as a trait: The development and validation of the Body Image States Scale. Eat Disord J Treat Prev. 2002; 10: 103–13. doi: [10.1080/10640260290081678](https://doi.org/10.1080/10640260290081678)
- **Spanish version:** Translated by the authors.

**************************************

The questionnaires constructed by the authors are included below:

1. **Emotions’ Scale (ES)**

**(Spanish translation:** Escala de emociones**)**

1. **Scale of appraisal of the body descriptions**

**(Spanish translation:** Escala de valoración de las descripciones del cuerpo**)**

**Emotions’ scale (ES) – English version**

Next, you will find different words that express an emotion. You should indicate to what extent you feel each one of these emotions RIGHT NOW, taking into account the following scale:

|  | **Not at all** | **Very little** | **A little** | **Some** | **Quite** | **A lot** | **Completely** |
| --- | --- | --- | --- | --- | --- | --- | --- |
| **Self-confidence** | 1 | 2 | 3 | 4 | 5 | 6 | 7 |
| **Insecurity** | 1 | 2 | 3 | 4 | 5 | 6 | 7 |
| **Anxiety** | 1 | 2 | 3 | 4 | 5 | 6 | 7 |
| **Anger** | 1 | 2 | 3 | 4 | 5 | 6 | 7 |
| **Disgust** | 1 | 2 | 3 | 4 | 5 | 6 | 7 |
| **Shame** | 1 | 2 | 3 | 4 | 5 | 6 | 7 |
| **Happiness** | 1 | 2 | 3 | 4 | 5 | 6 | 7 |
| **Sadness** | 1 | 2 | 3 | 4 | 5 | 6 | 7 |

**Escala de emociones (ES) – Spanish version**

A continuación, encontrarás una serie de palabras que expresan una emoción. Indica en qué medida sientes cada una de estas emociones en ESTE MOMENTO, teniendo en cuenta la siguiente escala:

|  | **Nada en absoluto** | **Muy poco** | **Poco** | **Algo** | **Bastante** | **Mucho** | **Totalmente** |
| --- | --- | --- | --- | --- | --- | --- | --- |
| **Autoconfianza** | 1 | 2 | 3 | 4 | 5 | 6 | 7 |
| **Inseguridad** | 1 | 2 | 3 | 4 | 5 | 6 | 7 |
| **Ansiedad** | 1 | 2 | 3 | 4 | 5 | 6 | 7 |
| **Enfado** | 1 | 2 | 3 | 4 | 5 | 6 | 7 |
| **Repulsión** | 1 | 2 | 3 | 4 | 5 | 6 | 7 |
| **Vergüenza** | 1 | 2 | 3 | 4 | 5 | 6 | 7 |
| **Felicidad** | 1 | 2 | 3 | 4 | 5 | 6 | 7 |
| **Tristeza** | 1 | 2 | 3 | 4 | 5 | 6 | 7 |

**Scale of appraisal of the body descriptions – English version**

In this questionnaire you will have to appraise the two comments you have made of each part of your body as “negative”, “neutral” or “positive”. You should rate it as you consider. There are no right or wrong answers.

|  | **Negative** |  |  |  |  | **Neutral** |  |  |  |  | **Positive** |
| --- | --- | --- | --- | --- | --- | --- | --- | --- | --- | --- | --- |
| **1a. For me, that my hair is…** | -5 | -4 | -3 | -2 | -1 | 0 | 1 | 2 | 3 | 4 | 5 |
| **1b. For me, that my hair is…** | -5 | -4 | -3 | -2 | -1 | 0 | 1 | 2 | 3 | 4 | 5 |
| **2a. For me, that my skin is…** | -5 | -4 | -3 | -2 | -1 | 0 | 1 | 2 | 3 | 4 | 5 |
| **2b. For me, that my skin is…** | -5 | -4 | -3 | -2 | -1 | 0 | 1 | 2 | 3 | 4 | 5 |
| **3a. For me, that my eyes are…** | -5 | -4 | -3 | -2 | -1 | 0 | 1 | 2 | 3 | 4 | 5 |
| **3b. For me, that my eyes are…** | -5 | -4 | -3 | -2 | -1 | 0 | 1 | 2 | 3 | 4 | 5 |
| **4a. For me, that my nose is…** | -5 | -4 | -3 | -2 | -1 | 0 | 1 | 2 | 3 | 4 | 5 |
| **4b. For me, that my nose is…** | -5 | -4 | -3 | -2 | -1 | 0 | 1 | 2 | 3 | 4 | 5 |
| **5a. For me, that my mouth is…** | -5 | -4 | -3 | -2 | -1 | 0 | 1 | 2 | 3 | 4 | 5 |
| **5a. For me, that my mouth is…** | -5 | -4 | -3 | -2 | -1 | 0 | 1 | 2 | 3 | 4 | 5 |
| **6a. For me, that my neck is…** | -5 | -4 | -3 | -2 | -1 | 0 | 1 | 2 | 3 | 4 | 5 |
| **6b. For me, that my neck is…** | -5 | -4 | -3 | -2 | -1 | 0 | 1 | 2 | 3 | 4 | 5 |
| **7a. For me, that my arms are…** | -5 | -4 | -3 | -2 | -1 | 0 | 1 | 2 | 3 | 4 | 5 |
| **7b. For me, that my arms are…** | -5 | -4 | -3 | -2 | -1 | 0 | 1 | 2 | 3 | 4 | 5 |
| **8a. For me, that my chest is…** | -5 | -4 | -3 | -2 | -1 | 0 | 1 | 2 | 3 | 4 | 5 |
| **8b. For me, that my chest is…** | -5 | -4 | -3 | -2 | -1 | 0 | 1 | 2 | 3 | 4 | 5 |
| **9a. For me, that my waist is…** | -5 | -4 | -3 | -2 | -1 | 0 | 1 | 2 | 3 | 4 | 5 |
| **9b. For me, that my waist is…** | -5 | -4 | -3 | -2 | -1 | 0 | 1 | 2 | 3 | 4 | 5 |
| **10a. For me, that my hips are…** | -5 | -4 | -3 | -2 | -1 | 0 | 1 | 2 | 3 | 4 | 5 |
| **10b. For me, that my hips are…** | -5 | -4 | -3 | -2 | -1 | 0 | 1 | 2 | 3 | 4 | 5 |
| **11a. For me, that my buttocks are…** | -5 | -4 | -3 | -2 | -1 | 0 | 1 | 2 | 3 | 4 | 5 |
| **11b. For me, that my buttocks are…** | -5 | -4 | -3 | -2 | -1 | 0 | 1 | 2 | 3 | 4 | 5 |
| **12a. For me, that my thighs are…** | -5 | -4 | -3 | -2 | -1 | 0 | 1 | 2 | 3 | 4 | 5 |
| **12b. For me, that my thighs are…** | -5 | -4 | -3 | -2 | -1 | 0 | 1 | 2 | 3 | 4 | 5 |
| **13a. For me, that my knees are…** | -5 | -4 | -3 | -2 | -1 | 0 | 1 | 2 | 3 | 4 | 5 |
| **13b. For me, that my knees are…** | -5 | -4 | -3 | -2 | -1 | 0 | 1 | 2 | 3 | 4 | 5 |
| **14a. For me, that my calves are…** | -5 | -4 | -3 | -2 | -1 | 0 | 1 | 2 | 3 | 4 | 5 |
| **14b. For me, that my calves are…** | -5 | -4 | -3 | -2 | -1 | 0 | 1 | 2 | 3 | 4 | 5 |
| **15a. For me, that my feet are…** | -5 | -4 | -3 | -2 | -1 | 0 | 1 | 2 | 3 | 4 | 5 |
| **15b. For me, that my feet are…** | -5 | -4 | -3 | -2 | -1 | 0 | 1 | 2 | 3 | 4 | 5 |

**Escala de valoración de las descripciones del cuerpo– Spanish version**

En este cuestionario tendrás que valorar los dos comentarios que has realizado de cada parte de tu cuerpo como “negativo”, “neutro” o “positivo”. Tienes que puntuarlo tal y como tú lo consideras. No hay respuestas buenas ni malas.

|  | **Negativo** |  |  |  |  | **Neutro** |  |  |  |  | **Positivo** |
| --- | --- | --- | --- | --- | --- | --- | --- | --- | --- | --- | --- |
| **1a. Para mí, que mi pelo sea…** | -5 | -4 | -3 | -2 | -1 | 0 | 1 | 2 | 3 | 4 | 5 |
| **1b. Para mí, que mi pelo sea…** | -5 | -4 | -3 | -2 | -1 | 0 | 1 | 2 | 3 | 4 | 5 |
| **2a. Para mí, que mi piel sea…** | -5 | -4 | -3 | -2 | -1 | 0 | 1 | 2 | 3 | 4 | 5 |
| **2b. Para mí, que mi piel sea…** | -5 | -4 | -3 | -2 | -1 | 0 | 1 | 2 | 3 | 4 | 5 |
| **3a. Para mí, que mis ojos sean…** | -5 | -4 | -3 | -2 | -1 | 0 | 1 | 2 | 3 | 4 | 5 |
| **3b. Para mí, que mis ojos sean…** | -5 | -4 | -3 | -2 | -1 | 0 | 1 | 2 | 3 | 4 | 5 |
| **4a. Para mí, que mi nariz sea…** | -5 | -4 | -3 | -2 | -1 | 0 | 1 | 2 | 3 | 4 | 5 |
| **4b. Para mí, que mi nariz sea…** | -5 | -4 | -3 | -2 | -1 | 0 | 1 | 2 | 3 | 4 | 5 |
| **5a. Para mí, que mi boca sea…** | -5 | -4 | -3 | -2 | -1 | 0 | 1 | 2 | 3 | 4 | 5 |
| **5a. Para mí, que mi boca sea...** | -5 | -4 | -3 | -2 | -1 | 0 | 1 | 2 | 3 | 4 | 5 |
| **6a. Para mí, que mi cuello sea...** | -5 | -4 | -3 | -2 | -1 | 0 | 1 | 2 | 3 | 4 | 5 |
| **6b. Para mí, que mi cuello sea...** | -5 | -4 | -3 | -2 | -1 | 0 | 1 | 2 | 3 | 4 | 5 |
| **7a. Para mí, que mis brazos sean...** | -5 | -4 | -3 | -2 | -1 | 0 | 1 | 2 | 3 | 4 | 5 |
| **7b. Para mí, que mis brazos sean...** | -5 | -4 | -3 | -2 | -1 | 0 | 1 | 2 | 3 | 4 | 5 |
| **8a. Para mí, que mi pecho sea...** | -5 | -4 | -3 | -2 | -1 | 0 | 1 | 2 | 3 | 4 | 5 |
| **8b. Para mí, que mi pecho sea...** | -5 | -4 | -3 | -2 | -1 | 0 | 1 | 2 | 3 | 4 | 5 |
| **9a. Para mí, que mi cintura sea...** | -5 | -4 | -3 | -2 | -1 | 0 | 1 | 2 | 3 | 4 | 5 |
| **9b. Para mí, que mi cintura sea...** | -5 | -4 | -3 | -2 | -1 | 0 | 1 | 2 | 3 | 4 | 5 |
| **10a. Para mí, que mis caderas sean...** | -5 | -4 | -3 | -2 | -1 | 0 | 1 | 2 | 3 | 4 | 5 |
| **10b. Para mí, que mis caderas sean...** | -5 | -4 | -3 | -2 | -1 | 0 | 1 | 2 | 3 | 4 | 5 |
| **11a. Para mí, que mis glúteos sean...** | -5 | -4 | -3 | -2 | -1 | 0 | 1 | 2 | 3 | 4 | 5 |
| **11b. Para mí, que mis glúteos sean...** | -5 | -4 | -3 | -2 | -1 | 0 | 1 | 2 | 3 | 4 | 5 |
| **12a. Para mí, que mis muslos sean...** | -5 | -4 | -3 | -2 | -1 | 0 | 1 | 2 | 3 | 4 | 5 |
| **12b. Para mí, que mis muslos sean...** | -5 | -4 | -3 | -2 | -1 | 0 | 1 | 2 | 3 | 4 | 5 |
| **13a. Para mí, que mis rodillas sean...** | -5 | -4 | -3 | -2 | -1 | 0 | 1 | 2 | 3 | 4 | 5 |
| **13b. Para mí, que mis rodillas sean...** | -5 | -4 | -3 | -2 | -1 | 0 | 1 | 2 | 3 | 4 | 5 |
| **14a. Para mí, que mis pantorrillas sean...** | -5 | -4 | -3 | -2 | -1 | 0 | 1 | 2 | 3 | 4 | 5 |
| **14b. Para mí, que mis pantorrillas sean...** | -5 | -4 | -3 | -2 | -1 | 0 | 1 | 2 | 3 | 4 | 5 |
| **15a. Para mí, que mis pies sean...** | -5 | -4 | -3 | -2 | -1 | 0 | 1 | 2 | 3 | 4 | 5 |
| **15b. Para mí, que mis pies sean...** | -5 | -4 | -3 | -2 | -1 | 0 | 1 | 2 | 3 | 4 | 5 |
